# Generation and characterization of chicken monocyte-derived dendritic cells

**DOI:** 10.3389/fimmu.2025.1517697

**Published:** 2025-02-04

**Authors:** Elie Ngantcha Tatchou, Romane Milcamps, Guillaume Oldenhove, Bénédicte Lambrecht, Fiona Ingrao

**Affiliations:** ^1^ Service of Avian Virology and Immunology, Sciensano, Brussels, Belgium; ^2^ Laboratory of Immunobiology, Université Libre de Bruxelles, Gosselies, Belgium; ^3^ Molecular Virology Unit, de Duve Institute, Université Catholique de Louvain, Brussels, Belgium

**Keywords:** monocyte-derived dendritic cells, maturation, *in vitro*, lipopolysaccharide, Newcastle disease virus

## Abstract

**Introduction:**

Dendritic cells (DCs) play a crucial role in orchestrating immune responses by bridging innate and adaptive immunity. *In vitro* generation of DCs from mouse and human tissues such as bone marrow and peripheral blood monocytes, has been widely used to study their immunological functions. In chicken, DCs have mainly been derived from bone marrow cell cultures, with limited characterization from blood monocytes.

**Methods:**

The present study takes advantage of newly available chicken immunological tools to further characterize chicken monocyte-derived dendritic cells (MoDCs), focusing on their phenotype, and functions, including antigen capture and T-cell stimulation, and response to live Newcastle disease virus (NDV) stimulation.

**Results:**

Adherent chicken PBMCs were cultured with recombinant chicken granulocyte-macrophage colony-stimulating factor (GM-CSF) and interleukin-4 (IL-4), for 5 days, leading to the upregulation of putative CD11c and MHCII, markers of DC differentiation. Subsequent stimulation with lipopolysaccharide (LPS) or 24 h triggered phenotypic maturation of MoDCs, characterized by the increased surface expression of MHCII and co-stimulatory molecules CD80 and CD40, and elevated IL-12p40 secretion. This maturation reduced endocytic capacity but enhanced the allogenic stimulatory activity of the chicken MoDCs. Upon NDV stimulation for 6 h, MoDCs upregulated antiviral pathways, including retinoic acid-inducible gene I (RIG-I)-like receptors (RLRs), melanoma differentiation-associated protein 5 (MDA5) and laboratory of genetics and physiology 2 (LGP2), alongside increased production of type I interferons (IFNs), and the pro-inflammatory cytokines tumor necrosis factor-α (TNF-α), IL-1β, and IL-6. However, these responses were downregulated after 24 hours.

**Conclusion:**

These findings provide a comprehensive characterization of chicken MoDCs and suggest their potential as a model for studying host-pathogen interactions.

## Introduction

Dendritic cells are uniquely capable of capturing, processing, and presenting exogenous antigens to T cells, rendering them the most effective antigen-presenting cells (1, 2). In steady state, DCs generally reside in peripheral tissues as immature cells with high endocytic and phagocytic capacities, constantly detecting microbial invasion through various pattern recognition receptors (PRRs) such as Toll-like receptors (TLRs), retinoic acid-inducible gene-I (RIG-I)-like receptors (RLRs), and C-type lectin receptors (CLRs). PRRs activation by “danger signals,” either associated with pathogens or directly derived from tissue injury damage-associated molecular patterns, triggers DC maturation. During this process, DCs undergo significant morphological changes, reduce their ability to uptake and process antigens, upregulate the expression of major histocompatibility complex (MHC) molecules, together with CD40, CD80, and CD86 costimulatory molecules, and produce cytokines that enhance and modulate immune responses (3, 4). Maturation is also associated with DC migration through afferent lymph vessels into the T-cell areas of secondary lymphoid organs, guided by the chemokine receptor CCR7, whose expression is upregulated in mature DCs (5–7). Consequently, mature DCs are highly efficient in antigen presentation in the context of MHC classes I (MHCI) and II (MHCII) molecules, leading to the priming of antigen-specific naïve CD8+ and CD4+ T cells respectively (2, 8, 9). Mature DCs are essential as accessory cells in generating primary antibody responses (10). Therefore, the activation of DCs is crucial for initiating the adaptive immune response.

Isolating large amounts of DCs can be challenging due to their low abundance in the peripheral blood and tissues. To address this limitation, methods have been developed in humans to generate large numbers of DCs by culturing precursor cells such as CD34+ bone marrow progenitor cells and CD14+ peripheral blood monocytes, in the presence of growth factors, particularly granulocyte-macrophage colony-stimulating factor (GM-CSF) and interleukin-4 (IL-4) (11–14). Human monocyte-derived DCs (MoDCs) have been widely used in various applications, including for testing vaccine and adjuvant immunogenicity and assessing compounds with immunosensitizing effects (15–20).

In chickens, the *in vitro* generation of functional DCs was first demonstrated by culturing bone marrow cells supplemented with chicken GM-CSF and IL-4 (21). The resulting chicken bone marrow-derived DCs (BmDCs) exhibited the typical morphology of DCs and expressed high levels of MHCII and putative CD11c, moderate levels of CD40, low levels of CD86, and no expression of DEC205, a CLR that has been shown to be abundantly expressed by mouse and human DCs (21–23). In addition, immature chicken BmDCs also expressed chemokine receptors 6 and 7 (CCR6 and CCR7) (24). Upon activation with lipopolysaccharide (LPS) or CD40 ligand (CD40L), the expressions of CD40, CD86, and DEC205 were upregulated (21). Furthermore, LPS-stimulated chicken BmDCs showed a decreased capacity to uptake FITC-dextran and fluorescent beads and displayed an enhanced ability to stimulate both allogenic and syngeneic Mixed Lymphocyte Reaction (MLR), as well as increased expression of Th1-promoting gamma interferon (IFN-γ) and interleukin-12 (IL-12) (21). The activation of chicken BmDCs is also accompanied by the downregulation of CCR6 and upregulation of chicken CCR7 expression (24). A recent study revealed that the chicken BmDC cultures are heterogeneous and consist of MHCII^low^ and MHCII^high^ cell subsets, corresponding to DCs at different maturation states (25). The MHCII^high^ subset of chicken BmDCs exhibited a more mature phenotype, characterized by higher surface expression of CD40 and CD80 and upregulation of mRNA levels of CCR7 and CD83. Furthermore, this subset showed increased expression of MRC1LB, the chicken homolog of mammalian mannose receptor c-type 1 (MRC1; also known as CD206) (25, 26). Additional studies have demonstrated that chicken BmDCs mature upon stimulation with highly pathogenic avian influenza viruses (HPAIV) (27) and inactivated infectious bursal disease virus (IBDV) (28).

This present study aimed to characterize the phenotype and function of under-studied chicken MoDCs. Unlike BmDCs, DCs derived from chicken peripheral blood monocytes offer the advantage of being a more accessible and minimally invasive source of cells. Chicken BmDCs and MoDCs have previously been generated and the differences in surface marker, TLRs, cytokine, and chemokine gene expression patterns have been examined between both cell populations following LPS stimulation (29). Upon LPS stimulation, the mRNA levels of surface markers CD40, CD80, CD83, CD86, and MHCII, pro-inflammatory cytokines IL-1b, IL-6, and TNF-α, chemokine CXCli2, and TLR4 and TLR15 were significantly upregulated in mature MoDCs. Similarly, gene expression analysis has been used to assess the differential responses of chicken MoDCs exposed to low pathogenic avian influenza viruses (LPAIV), HPAIV (30), *Salmonella Gallinarum*, and *Salmonella Typhimurium* (31).

Here, an in-depth analysis of chickens MoDCs was performed to evaluate their abilities in antigen capture and T-cell activation, and investigate their antiviral responses to Newcastle disease virus (NDV) stimulation.

## Materials and methods

### Chickens

Specific pathogen-free (SPF) White Leghorn chickens hatched from eggs purchased from VALO BioMedia™ (Osterholz-Scharmbeck, Germany). All birds were housed in biosecurity level 3 isolators (Montair, The Netherlands), with feed and water provided ad libitum.

### Generation and maturation of chicken monocyte-derived dendritic cells

Blood was collected under aseptic conditions from the brachial wing vein of 10- to 16-week-old SPF chickens and diluted with an equal volume of RPMI 1640 medium (Gibco, 52400025) containing heparin (Sigma-Aldrich, H-0878) at a concentration of 1000 units/mL of blood. Blood was transferred into 15 mL SepMate™ tubes (Stemcell Technologies, 85415) containing ficoll histopaque 1083 density gradient medium (Sigma-Aldrich, 10831) and centrifuged at 1200 × g for 10 min at room temperature (RT). The top layer was poured into 15 mL polypropylene conical tubes (BD, 352097), and the peripheral blood mononuclear cells (PBMCs) were washed twice in complete medium composed of RPMI medium supplemented with 10% heat-inactivated fetal bovine serum (FBS) (Biowest, S1860) and 50 µg/mL penicillin-streptomycin (Gibco, 15140122) by centrifugation at 300 × g for 8 min at RT. The cells were then resuspended in complete medium, and 2 × 10^7^ cells per well were seeded in sterile flat-bottomed 6-well culture plates (Nunclon™, 140675). Chicken blood monocytes were enriched by plastic adherence, by incubating PBMCs for two h at 41°C under 5% CO2. Non-adherent cells were then removed and discarded, and adherent cells were incubated for 5 days at 41°C in non-supplemented complete medium or complete medium supplemented with 10 ng/mL recombinant chicken granulocyte-monocyte colony-stimulating factor (GM-CSF) (KingFisher Biotech, RP0290C-025) and 10 ng/mL recombinant chicken interleukin-4 (IL-4) (KingFisher Biotech, RP0110C-025). The cells were incubated for 5 days at 41°C. On day 3, half of the culture medium was replaced with complete medium supplemented with GM-CSF and IL-4. On day 5, the culture medium was refreshed, and 500 ng/mL lipopolysaccharide (LPS) (Sigma-Aldrich, L4516) or 10 μg/mL live NDV LaSota strain (Lohmann Animal Health GmbH, Germany) was added for 6 or 24 h to induce cell maturation. Unstimulated cells were used as negative controls. The cells were harvested by gentle scraping with a cell scraper (TPP, 99002).

### Morphological analysis

The harvested cells were cytocentrifuged onto microscope slides for 5 min at 230 x g and fixed with 100% methanol (Merck, 1.06009.2511) for 3 min. After fixation, the slides were air-dried and stained for 20 min with Giemsa’s Azur eosin methylene blue staining solution (Sigma-Aldrich, 1.09204.0500) diluted 20X in deionized water. After incubation, the slides were washed twice with phosphate-buffered saline solution (PBS) (Gibco, 11503387) for 1 min and air-dried. The morphology of the differentiated MoDCs was examined by phase contrast microscopy, and images were obtained using the Leica Application Suite LAS V.4 program (Leica Microsystems Belgium BVBA, Diegem, Belgium).

### Flow cytometry

To characterize MoDCs, the cells were labeled with the antibodies listed in **Table 1**. Antibodies CD40, CD80, and DEC205 were purchased from the Veterinary Immunological Toolbox (Pirbright Institute), and the putative anti-chicken CD11c antibody (8F2 clone) antibody was kindly provided by Prof. Bernd Kaspers of the University of Munich. After stimulation, the cells were harvested, washed in PBS, and stained with fluorescence-conjugated specific primary antibodies or the corresponding isotype control in 50 µL of PBS for 30 min at 4°C. Indirect staining was performed to investigate the expression of CD11c, CD40, CD80, and DEC205. The cells stained with unlabeled primary antibodies were washed twice with PBS, centrifuged at 400 × g for 5 min, and labeled with fluorescence-conjugated secondary antibodies for 30 min at 4°C. The cells were then washed and fixed using BD Cytofix™ Fixation Buffer (Thermo Scientific, 554655). LIVE/DEAD^®^ Fixable Near-IR Dead Cell Stain Kit (Invitrogen, L34975) was used for dead cell exclusion, according to the manufacturer’s instructions. Events were acquired using a BD FACSVerse™ flow cytometer (BD Biosciences). Data analysis was performed using FlowJo™ software (version 10).

**Table 1:** List of antibodies.

Antibody	Isotype	Conjugate	Identifier	Source	Dilution
Mouse anti-chicken CD3 (Clone CT-3)	IgG1κ	PACBLU (Pacific BlueTM)	8200-26	Southern Biotech	20 µg/mL
Mouse anti-chicken CD4 (Clone EP97)	IgMκ	BIOT (Biotin)	8255-09	Southern Biotech	1 µg/mL
Mouse putative anti-chicken CD11c (Clone 8F2)	IgG1	UNLB (Unlabeld)	8F2	University of Munich	10 µg/mL
Mouse anti-chicken CD40 (Clone IG8)	IgG2a	UNLB	AV79	The Pirbright Institute	10 µg/mL
Mouse anti-chicken CD80 (Clone DC7)	IgG2a	UNLB	AV82	The Pirbright Institute	10 µg/mL
Mouse anti-chicken DEC205 (Clone FG9)	IgG1	UNLB	FG9	The Pirbright Institute	10 µg/mL
Mouse anti-chicken MHCII (Clone 2G11)	IgG1	FITC	8350-02	Southern Biotech	10 µg/mL
Mouse anti-chicken MRC1LB (Clone KUL01)	IgG1	AF647 (Alexa Fluor^®^ 647)	8420-31	Southern Biotech	2 µg/mL
Mouse IgG1 isotype control (Clone P3.6.2.8.1)	IgG1	UNLB	14-4714-82	eBioscience	5 µg/mL
Mouse IgG2a isotype control (Clone eBM2a)	IgG2a	UNLB	14-4724-82	eBioscience	5 µg/mL
Goat anti-mouse IgG1 (Polyclonal)	Goat IgG	FITC	1070-02	Southern Biotech	10 µg/mL
Goat anti-mouse IgG2a (Polyclonal)	Goat IgG2	AF647 (Alexa Fluor^®^ 647)	1082-31	Southern Biotech	5 µg/mL

### Quantification of cytokine production

Supernatants of unstimulated MoDCs and LPS- or live NDV-stimulated MoDCs were harvested after 6 or 24 h of culture and stored at -20°C until analysis. IL-12p40 concentrations were determined by sandwich ELISA using the chicken IL-12 p40 ELISA Kit (Invitrogen, ECH4RB) according to the manufacturer’s instructions.

### FITC-dextran uptake assay

The endocytic activity of unstimulated and LPS- or NDV-stimulated MoDCs was assessed using flow cytometry. After 6 or 24 h of stimulation, 2 x 10^5^ MoDCs were incubated with 1 mg of 40 kDa molecular weight FITC-dextran (Sigma-Aldrich, FD40S) for 2 h at 41°C. Nonspecific FITC-dextran binding to the cell surface was assessed by incubating cells at 4°C for 1 h. The cells were washed with ice-cold PBS and fixed with BD Cytofix™ Fixation Buffer.

### Allogeneic mixed lymphocyte reaction assay

Chicken MoDCs were either left unstimulated or treated with LPS for 6 h and were used as stimulator cells. Cultured MoDCs were harvested, washed twice with PBS, counted, and resuspended in complete culture medium. Responder CD4+ T cells were isolated aseptically from the spleens of allogeneic chickens, and cell suspensions were prepared as described previously (32). CD4+ cell enrichment was performed using positive immunomagnetic cell separation. Splenocytes were stained with 1 μg/mL biotinylated anti-chicken CD4 antibody (Southern Biotech, 8255-08) for 30 min at 4°C, labeled with 50 μL of Streptavidin Particles Plus – DM (BD IMag™, 557812) for 30 min at 4°C, and separated using a cell separation magnet (BD IMag™, 552311) according to the manufacturer’s instructions.

To assess cell proliferation, 2 × 10^7^ cells/mL were labeled with 5 μM 5,6-carboxyfluorescein diacetate succinimidyl ester (CFSE) (BD Horizon™, 565082) at RT for 10 min and washed twice with PBS containing 5% heat-inactivated FBS. MLR was performed by co-culturing 2 x 10^4^ stimulator MoDCs with 2 × 10^5^ responder CD4+ cells in 96-well tissue culture plates (stimulator: responder cells ratio of 1:10) for 5 days at 41°C. CFSE-labeled cells unstimulated or stimulated with 10 μg/mL Concanavalin A (ConA) (Sigma-Aldrich, C5275-5MG) were used as negative and positive controls, respectively. The proliferation of T cells was determined using CFSE dilution gated on live CD3+ cells.

### RNA extraction and real-time reverse transcription-PCR

MoDC RNA extraction, cDNA synthesis, and analysis of the relative expression of TNFα, IL-6, IL-1β (33), MDA5, LGP2, IFN-α, and IFN-β were performed according to a previously published protocol (34). The stability of three reference genes was checked in the 30 samples, and the data obtained was analyzed using GeNorm (CellCarta). RT-PCR data were normalized against the three most stable genes, i.e., *hprt1*, *hmbs*, and *tbp* (35). Normalized gene expression was quantified as the fold change relative to non-stimulated MoDCs at each time point.

### Statistical analyses

All statistical analyses were performed using GraphPad Prism version 9.5.0 (San Diego, CA, USA). Experimental groups and conditions were compared using a two-tailed Paired Student’s t-test and One-way ANOVA test. If normality and homogeneity of variance were not demonstrated, the non-parametric Wilcoxon matched-pairs signed and Friedman tests were used. Statistical significance was set at p < 0.05. The asterisks in the figures indicate statistical significance (*p<0.05, **p<0.01, ***p<0.001, and ****p<0.0001).

## Results

### The culture of adherent chicken PBMCs in the presence of GM-CSF and IL-4 induces CD11c and MHCII expression

**Figure 1:**
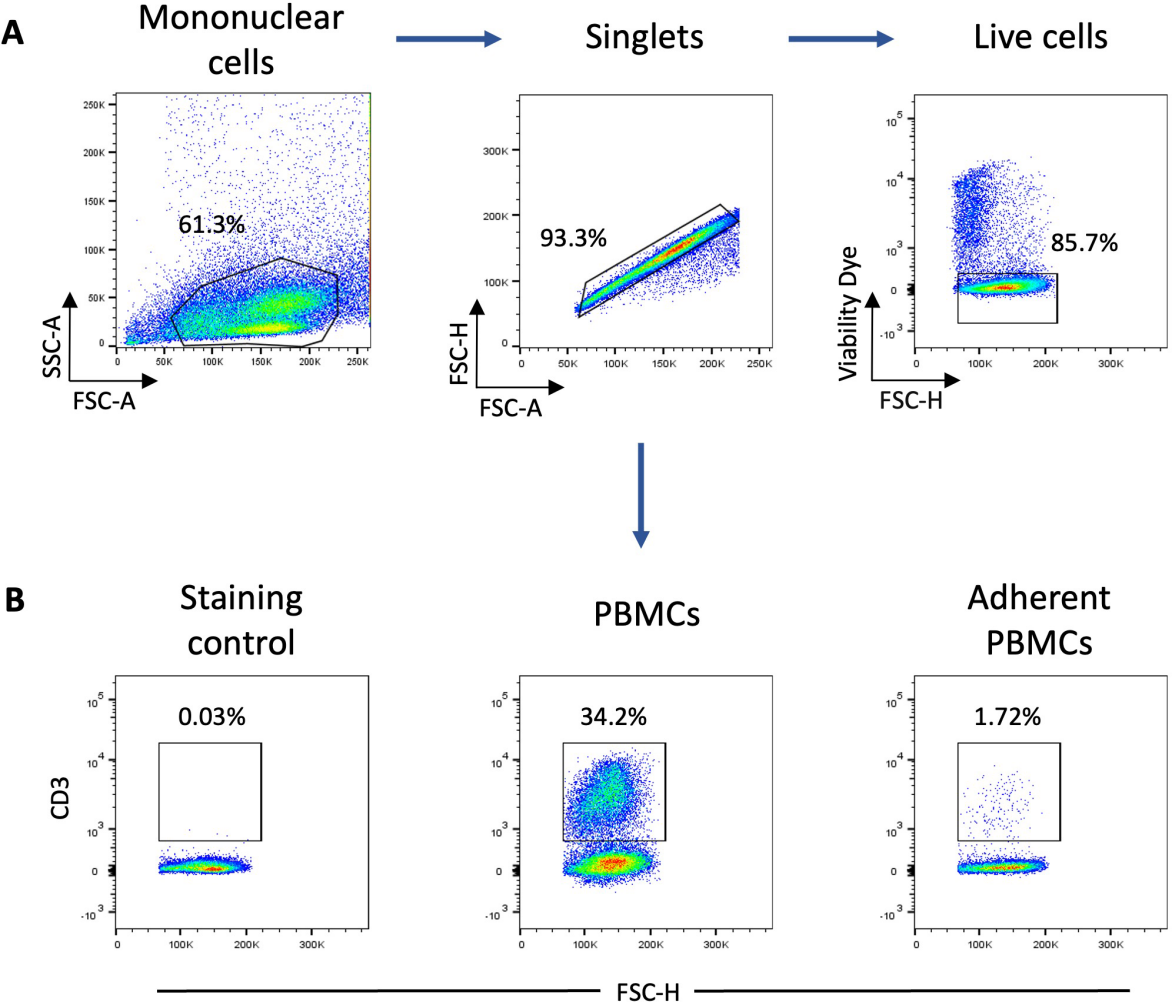
Analysis of monocyte population enrichment following plastic adherence of PBMCs. **(A)** Gating strategy for flow cytometry analysis of CD3+ and MRC1LB+ cells. The mononuclear cell population was gated based on forward scatter (FSC)-A and side scatter (SSC)-A parameters and singlets were selected from the FSC-A versus FSC-H. Dead cells were excluded using a viability dye **(B)** Representative flow cytometry dot plots of CD3 expression by live PBMCs and adherent PBMCs (n = 3). Numbers indicate the percentage of positive cells.

**Figure 2:**
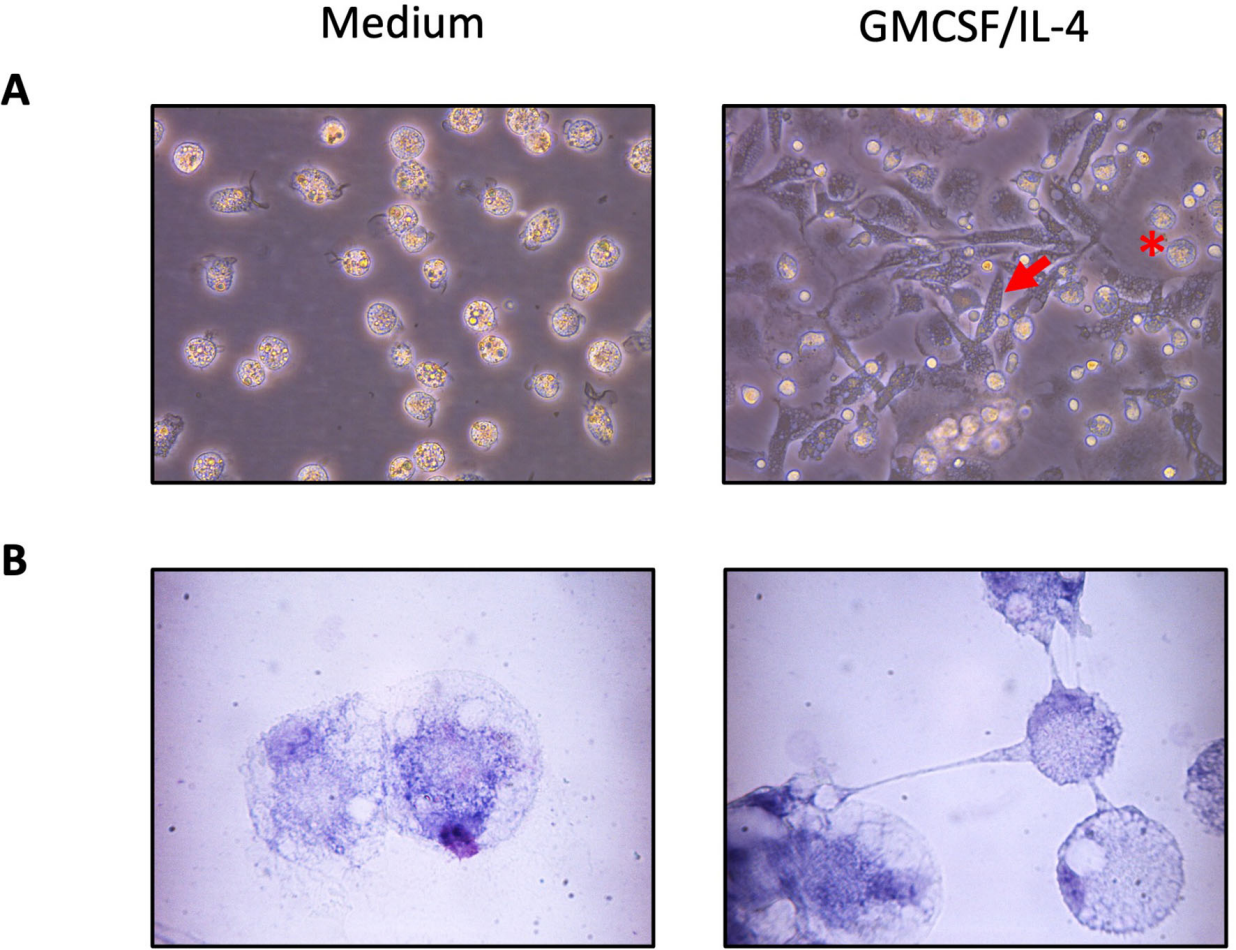
Morphology of chicken adherent PBMCs cultured 5 days in the presence of GM-CSF and IL-4. Representative images are of adherent cells cultured with non-supplemented complete medium (left) or supplemented with GM-CSF and IL-4 (right) at **(A)** 40x and **(B)** 100X magnification. The red arrows point to elongated differentiated DCs, and the red star indicates round cell morphology characteristic of monocytes.

**Figure 3:**
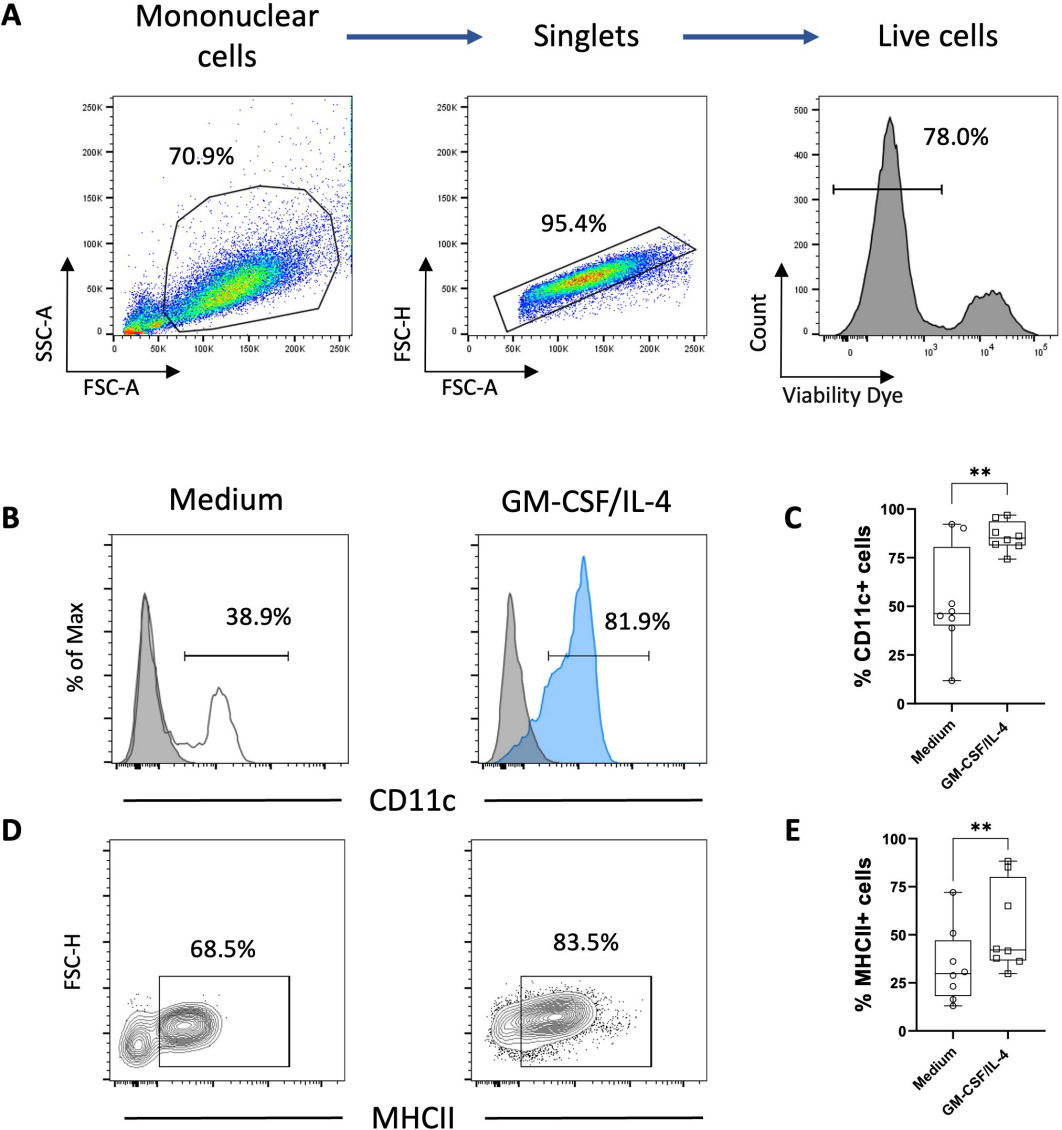
Phenotype analysis of chicken adherent PBMCs cultured 5 days in presence of GM-CSF and IL-4. **(A)** Gating strategy for flow cytometry analysis of chicken MoDCs based on FSC and SSC properties and singlets were selected from the FSC-A versus FSC-H. Dead cells were excluded using a viability dye. **(B)** Representative histograms of putative CD11c expression in adherent PBMCs incubated for 5 days only with non-supplemented complete medium (white) or in the presence of GM-CSF and IL-4 (blue). The background staining was evaluated using an isotype control (grey). Numbers on histograms represent the percentage of putative CD11c+ among live cells. **(C)** Boxplot of the percentage of putative CD11c+ cells among viable cells. Each symbol (circle or square) represents an individual chicken in the corresponding condition (n = 8). **p < 0.005, two-tailed paired t-test. **(D)** Representative contour plots of MHCII expression in adherent PBMCs incubated for 5 days with non-supplemented complete medium or in the presence of GM-CSF and IL-4. Numbers represent the percentage of MHCII+ cells among viable cells. **(E)** Boxplot of the percentage of MHCII+ cells among viable cells. Each symbol (circle or square) represents an individual chicken in the corresponding condition (n=8). A non-parametric Wilcoxon matched-pairs signed rank test was used to determine statistical differences (**p < 0.005).

One property of monocytes is to adhere spontaneously to plastic surfaces, unlike lymphocytes (36). We utilized this property to enrich our monocyte culture. Following this enrichment, the average percentage of T lymphocytes assessed by flow cytometry decreased from 25% to 1.5% (**Figure 1**). Monocyte-enriched PBMCs were then cultured in the presence of GM-CSF and IL-4 to promote their differentiation into MoDCs. After 5 days of culture, large loosely adherent cells with cytoplasmic protrusions, typical cytological features of DCs, were observed, while cells cultured without GM-CSF and IL-4 remained adherent and showed a round morphology (**Figures 2A, B**). Of note, cells in both conditions seemed to exhibit macropinocytotic vacuoles (macropinosomes), suggesting that macropinocytosis occurs in both cell types. Evaluation of cell viability at day 5 showed a significantly higher percentage of viable cells in monocyte-enriched PBMC cultures supplemented with GM-CSF and IL-4 compared to untreated cells (**Supplementary Figure S2**). Furthermore, these cytokines promote the differentiation of cells expressing putative CD11c and MHCII, two markers classically used to define DCs (**Figures 3A–E**).

### LPS-stimulation of chicken MoDCs increases MHCII and co-stimulatory molecules expression

**Figure 4:**
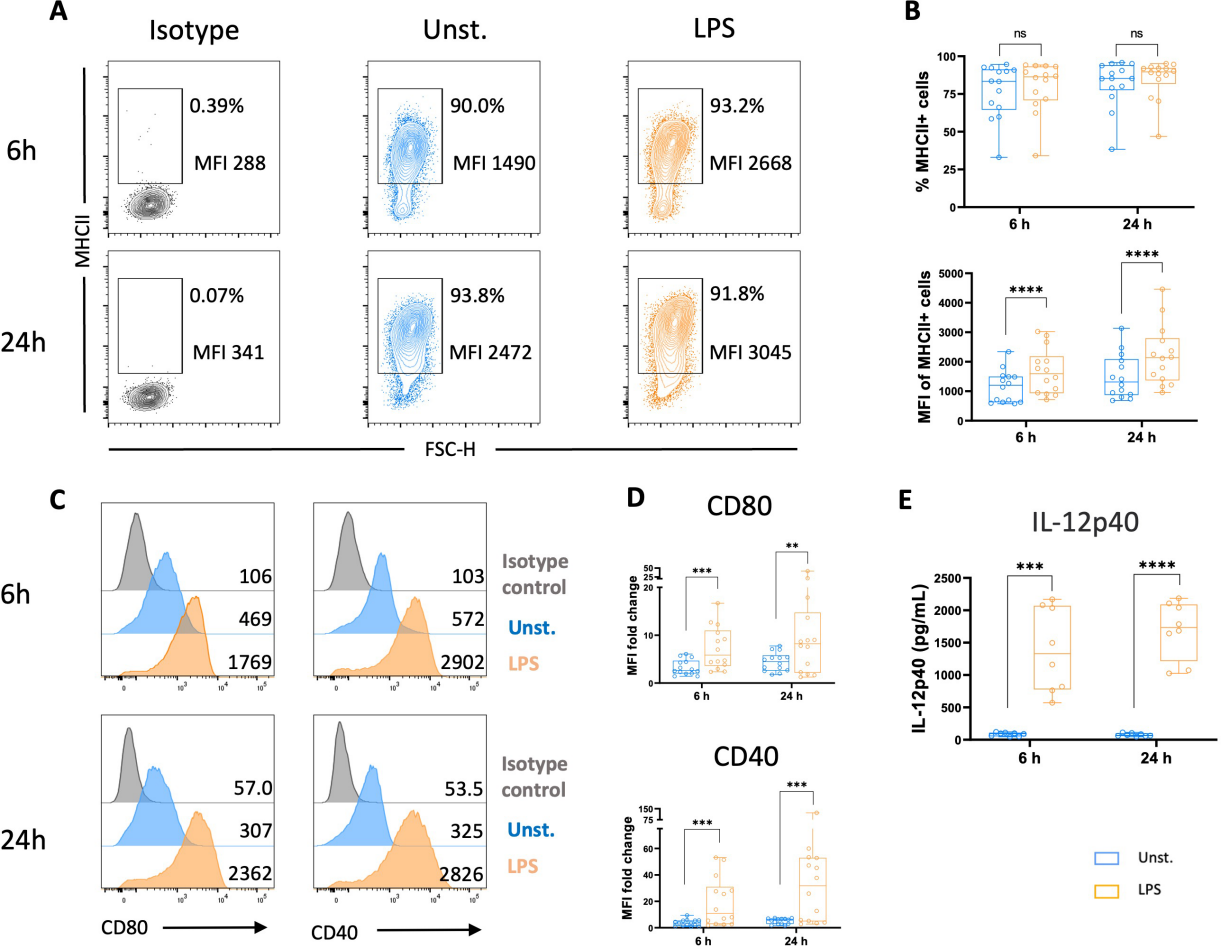
LPS stimulation increased MHCII and co-stimulatory molecules expressions, and IL-12p40 secretion. Flow cytometry was used to analyze unstimulated and LPS-stimulated MoDCs for their expression of MHCII, CD80, and CD40 after 6 and 24 h **(A)** Representative contour plots of MHCII with numbers on plots representing the percentage of MHCII+ cells gated on live cells and the MFI of MHCII+ cells. **(B)** The upper panel represents the percentage of MHCII+ cells among viable cells. Each circle represents an individual chicken in the corresponding condition. A non-parametric Wilcoxon matched-pairs signed rank test was used to determine statistical differences (ns, not significant). The lower panel represents MHCII MFI determined on viable MHCII+ cells. Each circle represents an individual chicken in the corresponding condition. A two-tailed paired t-test was used to determine statistical differences (****p<0.0001). **(C)** Representative flow cytometry histograms of CD80 (left panel), and CD40 (right panel) expressions in unstimulated cells (blue), LPS-stimulated MoDCs (orange), and isotype control (grey) after 6 and 24 h Numbers on histograms indicate the MFI values of the corresponding marker. **(D)** Boxplot of CD80 (upper panel) and CD40 (bottom panel) expression measured as MFI normalized to the isotype control. Each circle represents an individual chicken in the corresponding condition. A two-tailed paired t-test and a non-parametric Wilcoxon matched-pairs signed rank test were used to determine statistical differences (**p<0.01; ***p<0.001). The data from 3 independent experiments are displayed (n=14). **(E)** The production of IL-12p40 was quantified in the supernatants of unstimulated or LPS-stimulated MoDCs at 6 and 24 h by ELISA. Data are presented as boxplots with individual chicken values represented by circles. A two-tailed paired t-test was used to determine statistical differences (***p<0.001; ****p<0.0001). Data from two independent experiments (n=8) are displayed.

Next, we investigated whether chicken MoDCs can mature following an inflammatory stimulus. At 5 days, cells were cultured with LPS, commonly used to trigger DC maturation. We found similar frequencies of cells expressing MHCII in unstimulated versus LPS-stimulated cultures. However, the analysis of the median fluorescence intensity (MFI) determined in MHCII+ cells increased in the presence of LPS (**Figures 4A, B**). Additionally, the expression of both co-stimulatory molecules CD80 and CD40 was significantly higher following LPS stimulation (**Figures 4C, D**), indicating a mature DC phenotype. Following TLR activation, DCs secrete inflammatory cytokines, including IL-12, to generate an adequate immune response against the invading pathogens (4). In this context, we quantified the production of IL-12p40, a common subunit of the bioactive cytokines IL-12 and IL-23, by ELISA. LPS-stimulated MoDCs produced high levels of IL-12p40 compared to unstimulated cells (**Figure 4E**).

### LPS-stimulation induces functional maturation of chicken MoDCs

**Figure 5:**
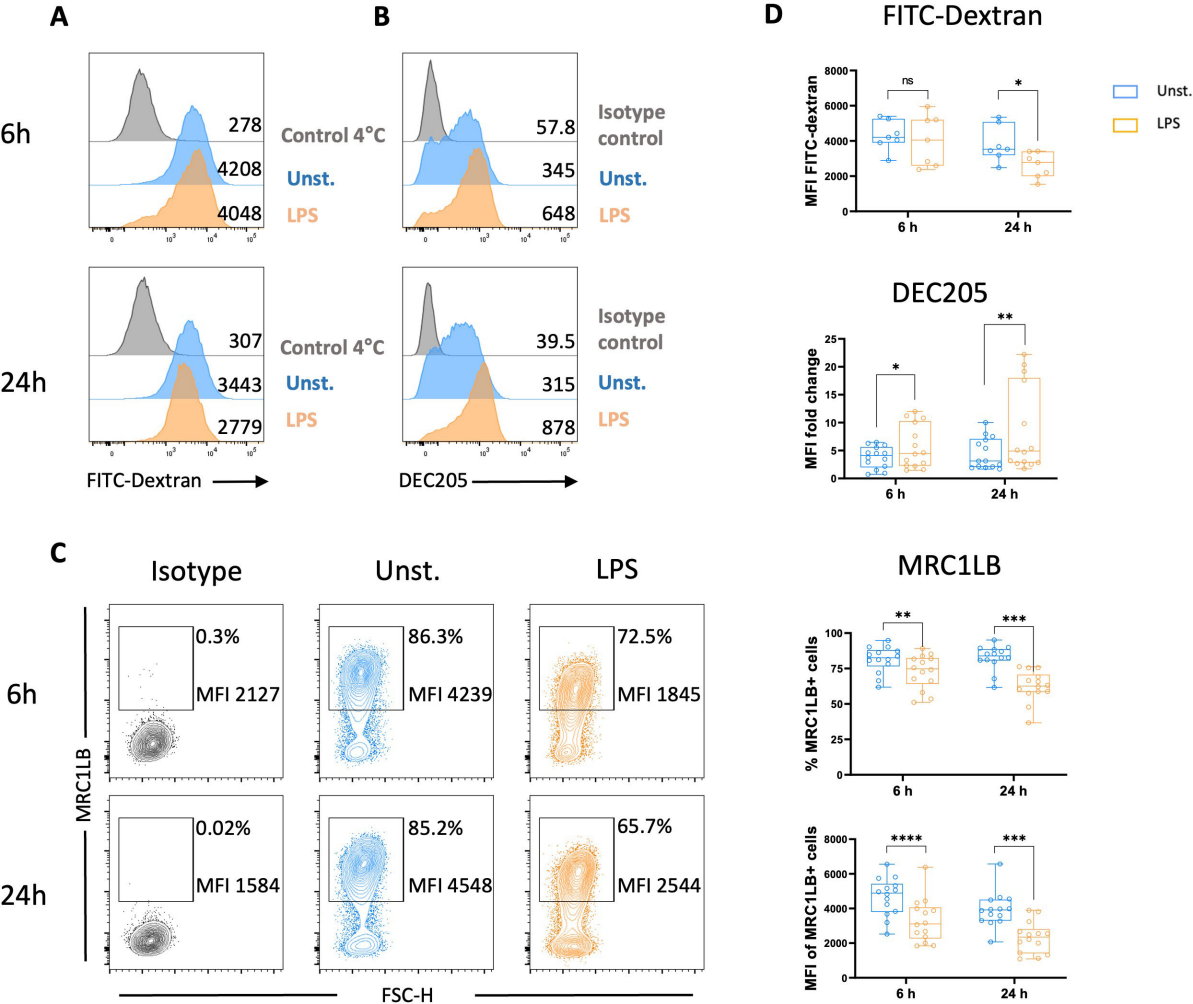
LPS stimulation decreased endocytic receptors expressions and endocytosis capacities. FITC-Dextran uptake and expression of receptors associated with endocytosis by unstimulated and LPS-stimulated MoDCs were analyzed after 6 and 24 h Representative flow cytometry histograms of FITC-dextran uptake **(A)** and DEC205 expression **(B)** of unstimulated (blue), LPS-stimulated (orange), and 4°C control/isotype control (grey). Numbers on histograms indicate the MFI values of the corresponding marker. **(C)** Representative contour plots of MRC1LB with numbers on plots representing the percentage of MRCLB+ cells gated on live cells and the MFI of MRC1LB+ cells. **(D)** Boxplot MFI of dextran-FITC+ cells, DEC205 expression measured as MFI normalized to the isotype control, the percentage of MRC1LB+ cells among live cells, MRC1LB MFI determined on viable MRC1LB+ cells are displayed. Each circle represents an individual chicken in the corresponding condition. A two-tailed paired t-test or a non-parametric Wilcoxon matched-pairs signed rank test (24 h) was used to determine statistical differences (*p<0.05; **p<0.01; ***p<0.001; ****p<0.0001); ns, not significant). The data from two independent experiments are displayed (n=14).

The ability of chicken MoDCs to mature following LPS stimulation was further characterized by evaluating their capacity for antigen capture by assessing FITC-dextran uptake. Chicken MoDCs stimulated for 24 h with LPS demonstrated significantly lower uptake of FITC-dextran than unstimulated chicken MoDCs, indicating a decrease in endocytic capacity. However, no difference in FITC-dextran uptake was observed between unstimulated and LPS-stimulated MoDCs at the 6-hour time point (**Figure 5A**). C-type lectins, such as MRC1 and DEC205, have been implicated in the uptake of carbohydrate-conjugated antigens by DCs. Additionally, the MRC1 has been identified as the major receptor responsible for MoDC FITC-dextran uptake (37). We next evaluated the expression of MRC1LB, the chicken homolog of human MRC1, and chicken DEC205 following the stimulation of chicken MoDCs with LPS. It was found that both the frequency of MRC1LB-expressing cells and MRC1LB MFI in MRC1LB+ cells were significantly reduced in LPS-stimulated chicken MoDCs compared to unstimulated controls (**Figures 5C, D**). In contrast, the C-type lectin receptor DEC205 was upregulated after LPS stimulation (**Figures 5B, D**).

**Figure 6:**
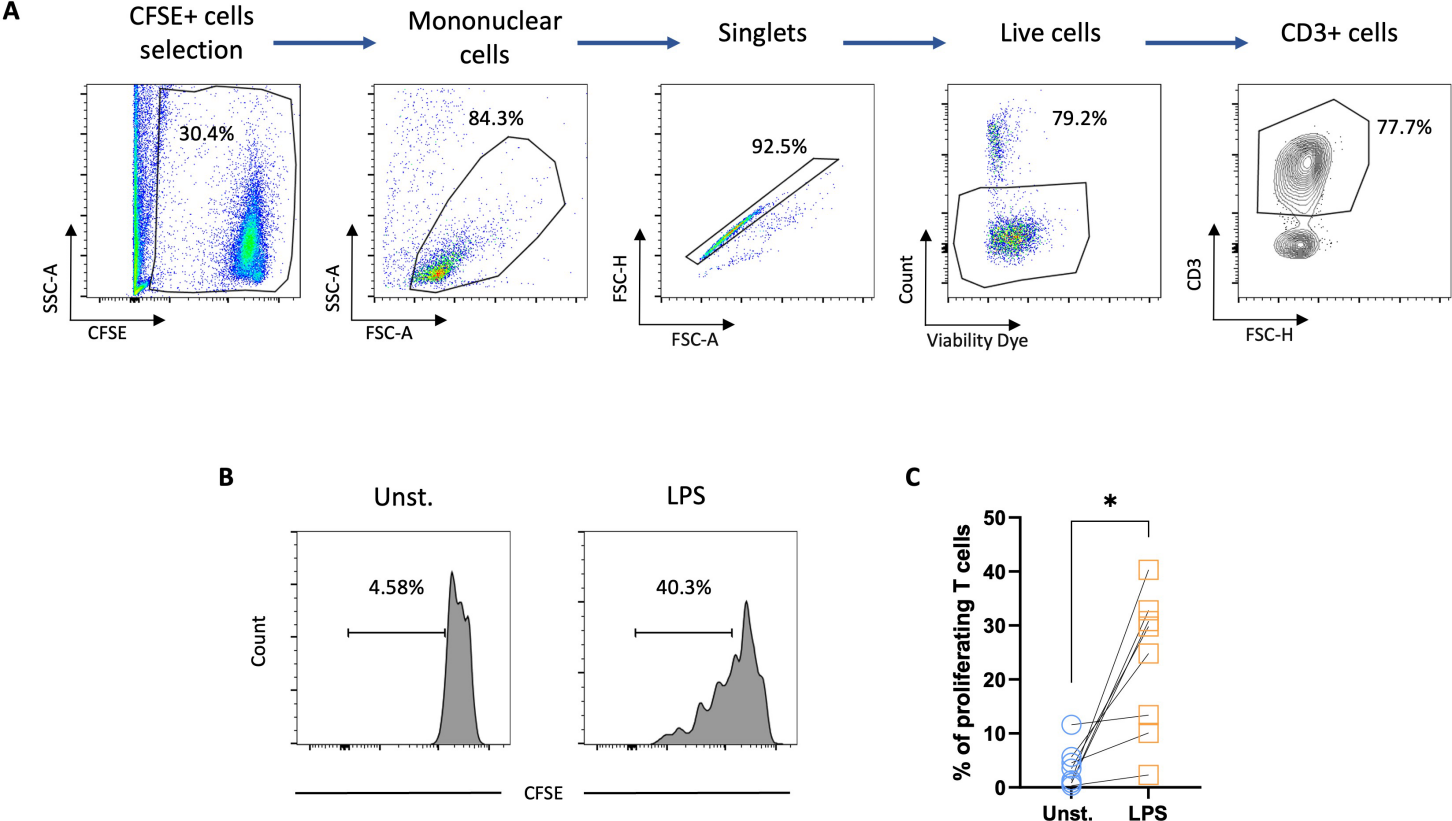
LPS-stimulated chicken MoDCs induced the proliferation of autologous T cells. Splenocytes from naïve chickens were isolated and CD4 T cells were enriched by magnetic selection. The CD4 T cells were then CFSE-labelled and co-cultured with allogeneic unstimulated or 6 h LPS-stimulated MoDCs. **(A)** Flow cytometry gating strategy. CFSE-stained cells were selected. The mononuclear cell population was gated based on FSC-A and SCC-A parameters and singlets were selected from the FSC-A versus FSC-H. Dead cells were excluded using a viability dye and CD3+ cells were gated based on CD3 versus FSC-H. **(B)** Representative histograms of unstimulated and LPS-stimulated CFSE-labeled T cells proliferation. Numbers on histograms indicate the percentage of proliferating T cells. **(C)** Proportions of proliferating T cells. A two-tailed paired t-test was used to determine statistical differences (*p<0.01). The data from two independent experiments are displayed (n=8).

The functional properties of GM-CSF/-IL-4–differentiated chicken MoDCs were finally characterized by assessing their ability to stimulate the proliferation of allogeneic T cells in a mixed lymphocyte reaction (MLR). CFSE-labeled allogeneic spleen CD4+ T cells were co-cultured with unstimulated or MoDCs stimulated with LPS for 6 h. The results showed a significant expansion of the T-cell population when cultured with LPS-stimulated MoDCs (**Figures 6A, B**), demonstrating their allostimulatory capacity.

### Newcastle disease virus promotes maturation and activates the antiviral program of chicken MoDCs

**Figure 7:**
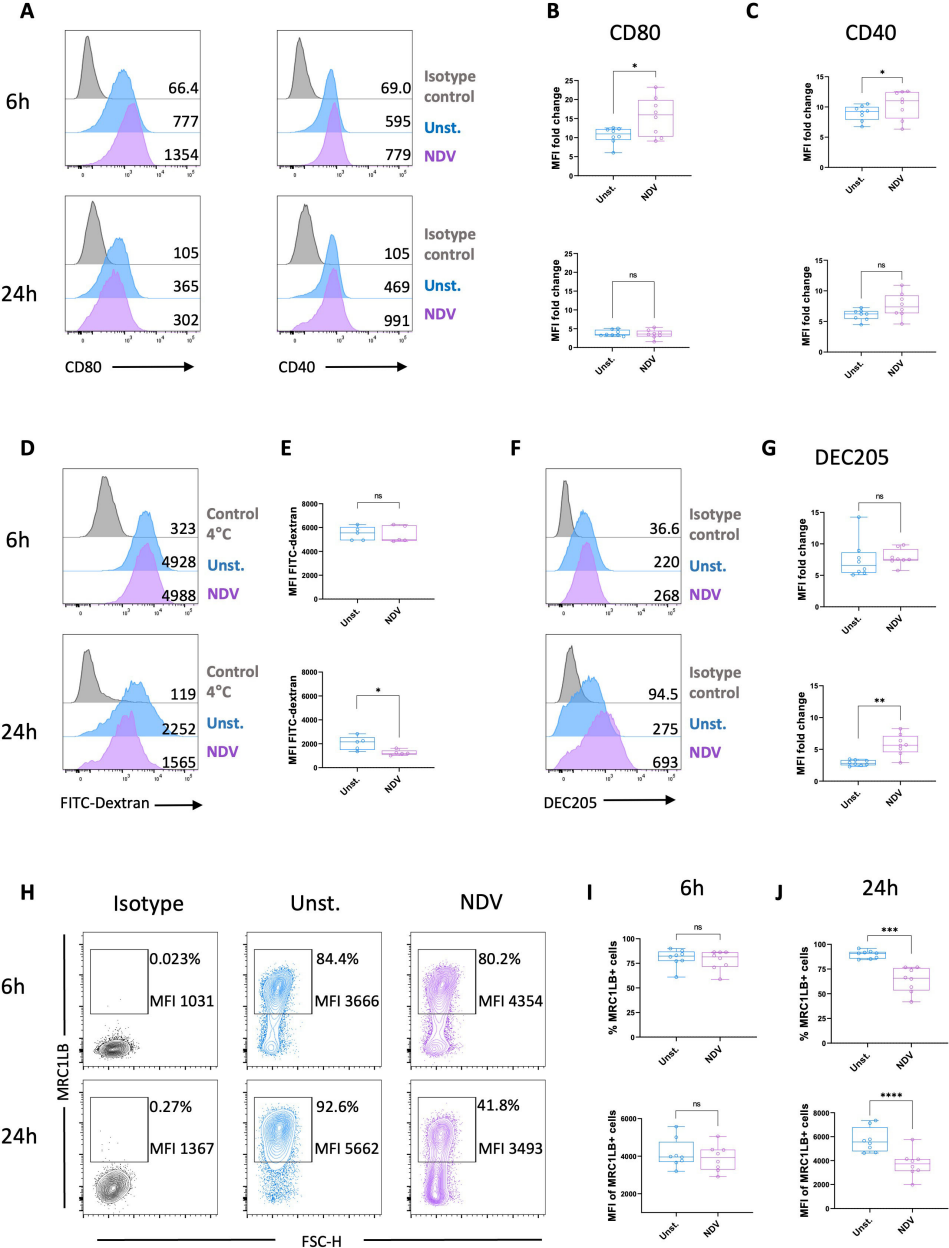
NDV stimulation induced maturation of chicken MoDCs. **(A)** Representative histograms of CD80 (left panel) and CD40 (right panel) expressions in unstimulated cells (blue), NDV-stimulated MoDCs (purple), and isotype control (grey) after 6 and 24 h Numbers on histograms indicate the MFI values of the corresponding marker. Boxplot of CD80 **(B)** and CD40 **(C)** expression measured as MFI normalized to the isotype control. Each circle represents an individual chicken in the corresponding condition. A two-tailed paired t-test and a non-parametric Wilcoxon matched-pairs signed rank test were used to determine statistical differences (*p<0.05; ns, not significant). The data from two independent experiments are displayed (n=8). **(D)** Representative histograms of dextran-FITC uptake of unstimulated (blue), NDV-stimulated (purple) MoDCs, and 4°C negative control (grey) after 6 h (upper panel) or 24 h stimulation (bottom panel). Numbers on histograms indicate the MFI of the fluorescent tracer. **(E)** Boxplot of dextran-FITC uptake measured as MFI after 6 h (upper panel) or 24 h stimulation (bottom panel). Each circle represents an individual chicken in the corresponding condition (n=5). A two-tailed paired t-test and a non-parametric Wilcoxon matched-pairs signed rank test were used to determine statistical differences (*p<0.05; ns, not significant). **(F)** Representative histograms DEC205 expression in unstimulated cells (blue), NDV-stimulated MoDCs (purple), and isotype control (grey) after 6 and 24 h Numbers on histograms indicate the MFI values of the corresponding marker. **(G)** Boxplot of DEC205 expression measured as MFI normalized to the isotype control. Each circle represents an individual chicken in the corresponding condition. A two-tailed paired t-test and a non-parametric Wilcoxon matched-pairs signed rank test were used to determine statistical differences (*p<0.05; **p<0.01; ns, not significant). Data from two independent experiments (n=8) are displayed. **(H)** Representative contour plots of MRC1LB expression. The numbers on the plots represent the percentage of MRC1LB+ cells gated on viable cells and the MFI of MRC1LB+ cells. Boxplot of the percentage of MRC1LB+ cells among viable cells and MFI determined on viable MRC1LB+ cells after 6 h **(I)** or 24h **(J)** NDV stimulation. Each circle represents an individual chicken in the corresponding condition. A two-tailed paired t-test and a non-parametric Wilcoxon matched-pairs signed rank test were used to determine statistical differences (***p<0.001; ****p<0.0001; ns, not significant). The data from two independent experiments are displayed (n=8).

To investigate the type of responses induced by MoDCs generated *in vitro* following stimulation by a chicken virus, cells were cultured with live NDV for 6 h and 24 h. Following a 6 h exposure to NDV, the chicken MoDCs exhibited a characteristic DC morphology indicated by the formation of non-adherent cell aggregates displaying multiple elongated cytoplasmic projections. These cell clusters became larger after 24h of NDV stimulation (**Supplementary Figure S5A**). Notably, chicken MoDCs viability remained comparable to that of the unstimulated control after culturing with NDV, whereas it was significantly reduced after 24h incubation with the virus (**Supplementary Figure S5B**). The expression of costimulatory molecules CD80 and CD40 was significantly upregulated after 6 h of stimulation with NDV (**Figures 7A–C**), which correlated with a slight increase in MHCII expression (**Supplementary Figure S7**). Additionally, chicken MoDCs exhibited a reduced FITC-Dextran uptake capacity, accompanied by a downregulation of MRC1LB, while DEC205 expression was upregulated at 24 h (**Figures 7D–J**). These results indicate that NDV-triggered chicken MoDCs undergo phenotypic and functional maturation.

**Figure 8:**
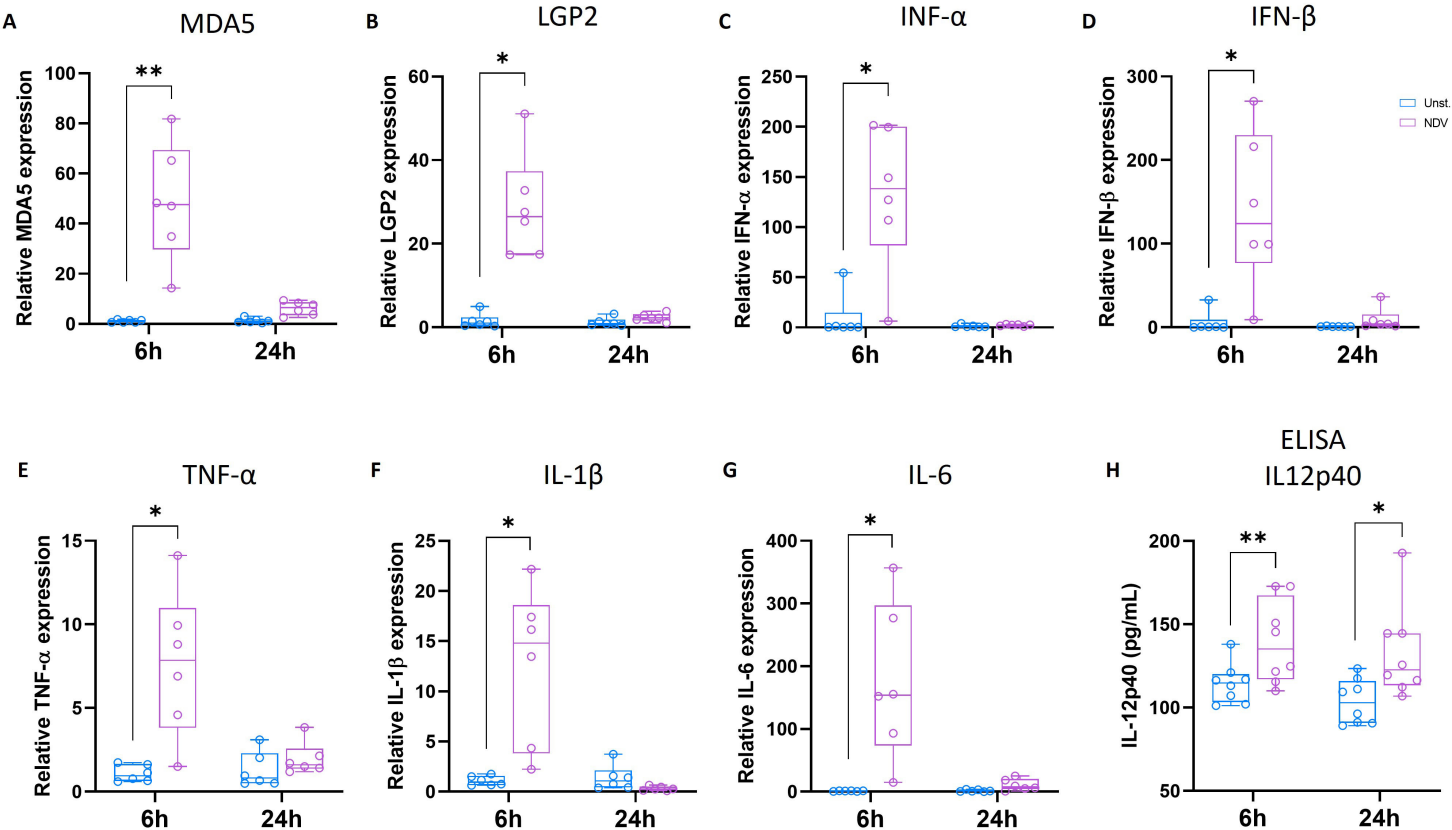
6 h NDV stimulation enhanced antiviral innate immune response. Chicken MoDC was generated and stimulated with NDV for 6 and 24 h Genes expression was analyzed by RT-PCR. Boxplots representing relative expression of MDA5 **(A)**, LGP2 **(B)**, IFN-α **(C)**, IFN-β **(D)**, TNF-α **(E)**, IL1-β **(F)**, and IL6 **(G)** determined in unstimulated (blue) and NDV-stimulated MoDCs (purple). Each circle represents an individual chicken in the corresponding condition. The data were normalized to HMBS, TBP, and HPRT1 expressions and calculated according to the 2^-ΔΔCT^ method. A two-tailed paired t-test and a non-parametric Wilcoxon matched-pairs signed rank test were used to determine statistical differences (*p<0.05; **p<0.01). The data from two independent experiments are displayed (n=6). **(H)** The production of IL-12p40 was quantified in the supernatants of unstimulated or NDV-stimulated MoDCs at 6 and 24 h by ELISA. Each circle represents an individual chicken. A two-tailed paired t-test and a non-parametric Wilcoxon matched-pairs signed rank test were used to determine statistical differences (*p<0.05; ns, not significant). Data from two independent experiments (n=8) are displayed.

Using qRT-PCR, we quantified the gene expression of several molecules involved in the antiviral response, including MDA5 and LGP2, which are cytosolic RNA helicases responsible for recognizing viral RNA, including NDV. In particular, MDA5 and LGP2 are known to play crucial roles in the induction of type I IFN (38, 39). A significant upregulation of MDA5 and LGP2 mRNA levels was observed 6 h post-NDV stimulation, while their expression was not significantly different from that observed in the unstimulated condition at 24 h (**Figures 8A, B**). Similarly, the expression of type I IFN-α and IFN-β mRNAs was significantly upregulated at 6 h in NDV-incubated MoDCs, with no statistical difference observed at 24 h (**Figures 8C, D**). The upregulation of type I IFN expression was confirmed by the significantly increased production of IFN-α protein in the supernatant of NDV-stimulated MoDCs at both 6 and 24 h (**Supplementary Figure S8**).

Upon activation by exposure to viral pathogens and bacterial products, DCs produce a range of cytokines that play crucial roles in modulating immune response. In this study, the expression of proinflammatory cytokines, including TNF-α, IL-1β, and IL-6, was analyzed in chicken MoDCs in response to 6 or 24 h of NDV stimulation using qRT-PCR. The mRNA levels of TNF-α, IL-1β, and IL-6 were significantly upregulated 6 h after NDV stimulation, but no differences were observed after 24 h of stimulation. (**Figures 8E–G**). Additionally, the production and secretion of IL-12p40 quantified by ELISA was significantly increased in the supernatants of both 6 and 24 h NDV-stimulated MoDCs (**Figure 8H**).

## Discussion

The main objective of this study was to deepen the phenotypic and functional characterization of chicken MoDCs. *In vitro*-generated chicken BmDCs have been previously defined by typical DC morphology, and MHCII and putative CD11c expression (21). In addition, chicken BmDCs were defined by their ability to undergo maturation upon stimulation, a key feature of DCs (3, 21, 25). Previous studies have evaluated the chicken MoDCs differentiation *in vitro* based on their morphology and the CD14 and CD83 mRNA levels (29, 31). Using similar culture protocol, this study demonstrated that adherent chicken PBMCs cultured with GM-CSF and IL-4 for five days displayed high surface expression of putative CD11c, using 8F2 antibody, and MHCII, along with typical DC morphology. Of note, although 8F2 is widely known as putative anti-chicken CD11c, it was recently suggested that its primary target is CD11d, which interacts with integrin beta 2 (CD18) (40). Our results are consistent with previous report on chicken BmDCs cultured with GM-CSF and IL-4 (21). These findings suggest that chicken MoDCs likely differentiate from monocytes within adherent PBMC populations, similarly to the previously demonstrated *in vitro* DC generation from human CD14+ monocytes (41–43).

The phenotypic characterization of chicken MoDCs maturation has so far been constrained by the limited availability of immunological tools such as monoclonal antibodies (44). Earlier studies have documented increased gene expression of MHCII or costimulatory molecules such as CD86, CD80, and CD40 following LPS stimulation (29, 31). Using newly available monoclonal antibodies, we found that LPS stimulation induced phenotypic maturation in chicken MoDCs, as evidenced by increased surface expression of MHCII and CD80 and CD40. These findings are consistent with prior reports showing increased surface expression of MHCII and costimulatory molecules on chicken BmDCs upon LPS stimulation (21). Additionally, the enhanced secretion of IL12p40, suggests that chicken MoDCs differentiated from peripheral blood cells can produce cytokines playing an important role in the regulation of T-cell activation (33, 45–48). Furthermore, DCs are known for their potent ability to stimulate naïve T cells in primary MLR (49, 50). LPS stimulation has been previously shown to enhance the allostimulatory capacity of chicken BmDCs (21). Similarly, recent findings have shown that the stimulation of chicken MoDCs with *Salmonella Typhimurium* significantly increased allogeneic chicken T-cell proliferation (31). Consistent with these findings, the present study revealed that LPS stimulation increased the allostimulatory capacity of chicken MoDCs, indicating functional maturation. LPS-stimulated chicken MoDCs also showed reduced FITC-dextran accumulation, reflecting a decrease in endocytic capacities, a hallmark of DC maturation (3). Endocytic receptors are typically downregulated during DC maturation (51, 52). The expression of the mannose receptor MRC1, a key receptor of FITC-dextran endocytosis, is downregulated in mature human MoDC (52). Due to its homology to mammalian MRC1, chicken MRC1L-B has been suggested to play a role in carbohydrate recognition and uptake (26). However, its involvement in dextran uptake has not been demonstrated. Here, downregulation of the surface expression of MRC1LB correlates with a reduction in FITC-dextran uptake capacity of LPS-stimulated chicken MoDCs. Interestingly, mature chicken MoDCs displayed a distinct upregulation of DEC205 expression pattern when compared to MRC1LB. This increase in DEC205 expression was previously reported at both protein and mRNA levels in mature human MoDCs, and it was postulated that DEC205 has a non-endocytic function in mature dendritic cells (22, 53, 54). Despite downregulation of endocytosis, the upregulation of DEC205 may also suggest that mature chicken MoDCs retain some capacity for antigen uptake, as previously observed in mouse BmDCs (55).

The antiviral response of chicken MoDCs was investigated through their infection with the NDV LaSota strain. In chickens, RNA viruses like NDV are sensed through the PPRs, including TLR3 and TLR7, and the RLRs such as MDA5, and LGP2 (56, 57). The activation of these pathways triggers type I IFN production and the release of pro-inflammatory cytokines (58). Previous studies indicated that the NDV LaSota strain triggered robust type I IFN and pro-inflammatory cytokine expressions *in vivo* in chickens (59) and *in vitro* in human tumor cell lines (60). In mature chicken BmDCs, 6 h of infection with NDV LaSota was shown to increase the expression of MDA5, LGP2, TLR3, TLR7, and type I IFN IFN-α and IFN-β (61). Unlike chicken BmDCs, little is known regarding the ability of chicken MoDCs to respond to NDV stimulation. In contrast, the effects of NDV on MoDCs have been characterized in humans due to its oncolytic properties (62). A recombinant NDV (rNDV) expressing GFP has been shown to induce maturation of human MoDCs (63). rNDV infection upregulated the expression of CD40, CD80, MHCI, and MHCII and increased the production of IFN-α, IL-6, and TNF-α (63). MEDI5395, a recombinant attenuated Newcastle disease virus engineered to express a human granulocyte-macrophage colony-stimulating factor transgene, was also shown to induce the activation of human MoDCs by upregulating MHC II, CD86, and CD83 (64). In addition, human MoDCs infected with MEDI5395 showed increased allogeneic T-cell activation (64). Consistent with these results, we showed that NDV stimulation for 6 h activated MDA5 and LGP2 in chicken MoDCs, leading to robust production of type I IFNs, and proinflammatory cytokines, including IL-12p40, IL-1, and IL-6. This response was, however, strongly downregulated after 24 h, which is in accordance with previous studies showing decreased gene expression of type I IFN-α and IFN-β and proinflammatory molecules IL-18, IL-6, and IL-8 in chicken BmDCs stimulated with NDV LaSota (61). Similar suppression of type I IFN and proinflammatory cytokines has been reported previously following NDV infection (65, 66). It has been suggested that the mechanism used by NDV to evade host detection involves the structural V protein, which promotes the degradation of key antiviral signaling molecules like MAVS and phospho-STAT1, thereby suppressing RLR-mediated type I IFN production (65, 66). Additionally, the V protein of various paramyxoviruses, including NDV, has been shown able to bind MDA5 and LGP2, preventing their activation and the subsequent production of type I IFN (67, 68). Additionally, NDV-stimulated MoDCs exhibited phenotypical changes indicative of T-cell activation capacity. Our findings align with previous reports of increased surface MHCII and costimulatory molecule expression upon NDV LaSota stimulation in human MoDCs (63) and murine BmDCs (69). However, after 24 h, the MHCII expression declined, along with a reduction in endocytic activity and MRC1LB expression. The observed downregulation of MHC II and antigen uptake capacity following activation supports the concept that during maturation, DCs prioritize antigen presentation over antigen capture, optimizing their ability to stimulate T-cells (70).

Taken together, these data suggest that adherent chicken peripheral blood cells cultured with GM-CSF and IL-4 differentiate into bona fide dendritic cells capable of maturing in response to microbial stimuli, with upregulated expression of molecules playing a critical role in regulating T-cell activation. Further studies could be carried out to examine gene expression profiles during various stages of chicken MoDCs differentiation from monocytes to immature MoDCs and in responses to diverse stimuli. The present study also highlights the advantages of using *in vitro* generated chicken MoDCs over *in vivo* models. The abundance of monocyte precursors in peripheral blood combined with minimally invasive isolation methods, offers an ethical and scalable approach for generating large number of chicken MoDCs. This model holds promise for studying host-pathogen interactions, evaluating pathogenicity of infectious organisms, and testing immunostimulatory agents such as vaccines and adjuvants.

## Data Availability

The raw data supporting the conclusions of this article will be made available by the authors, without undue reservation.
