# Supplementary material for: Generation and characterization of chicken monocyte-derived dendritic cells
Source: Front Immunol. 2025 Feb 4;16:1517697. doi: 10.3389/fimmu.2025.1517697 (PMC11832469; doi:10.3389/fimmu.2025.1517697)
Supplement: Supplementary file 1 [file DataSheet1.docx]

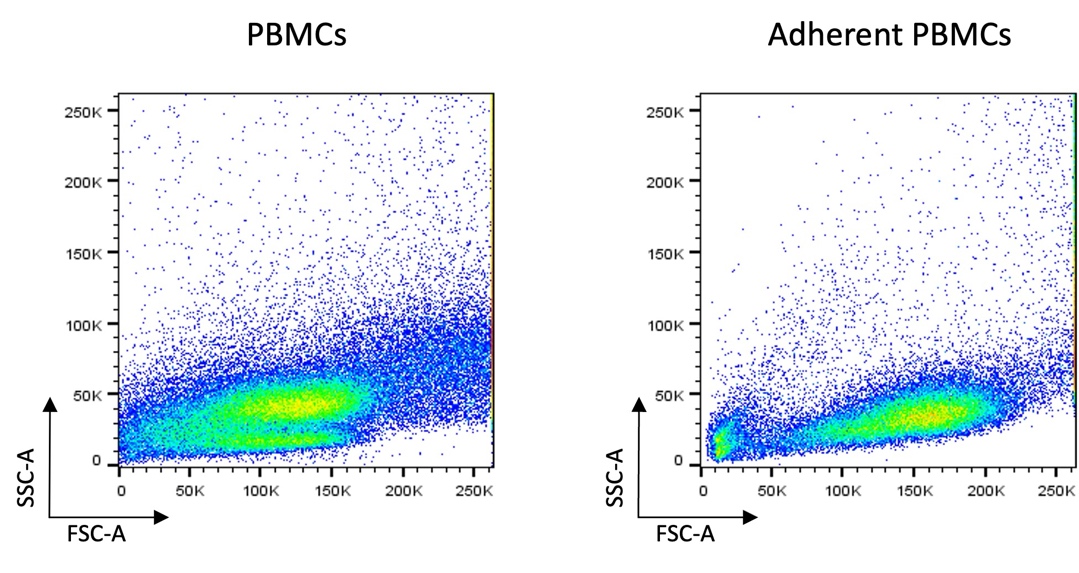


**Figure S1**. Analysis of FSC-SSC parameters enrichment following plastic adherence of PBMCs.

Representative ﬂow cytometry dot plots of forward scatter (FSC)-A and side scatter (SSC)-A of PBMCs and adherent PBMCs (n = 3).

**Figure S2.** Culture of monocyte-enriched chicken PBMCs with GM-CSF and IL-4 reduced cell mortality.

Cell viability was assessed by flow cytometry using a viability dye. Boxplot of the percentage of live cells among singlets in adherent PBMCs incubated for 5 days only with non-supplemented complete medium or in the presence of GM-CSF and IL-4. Each symbol (circle or square) represents an individual chicken in the corresponding condition (n = 8). A non-parametric Wilcoxon matched-pairs signed rank test was used respectively to determine statistical differences (**: p<0.01).


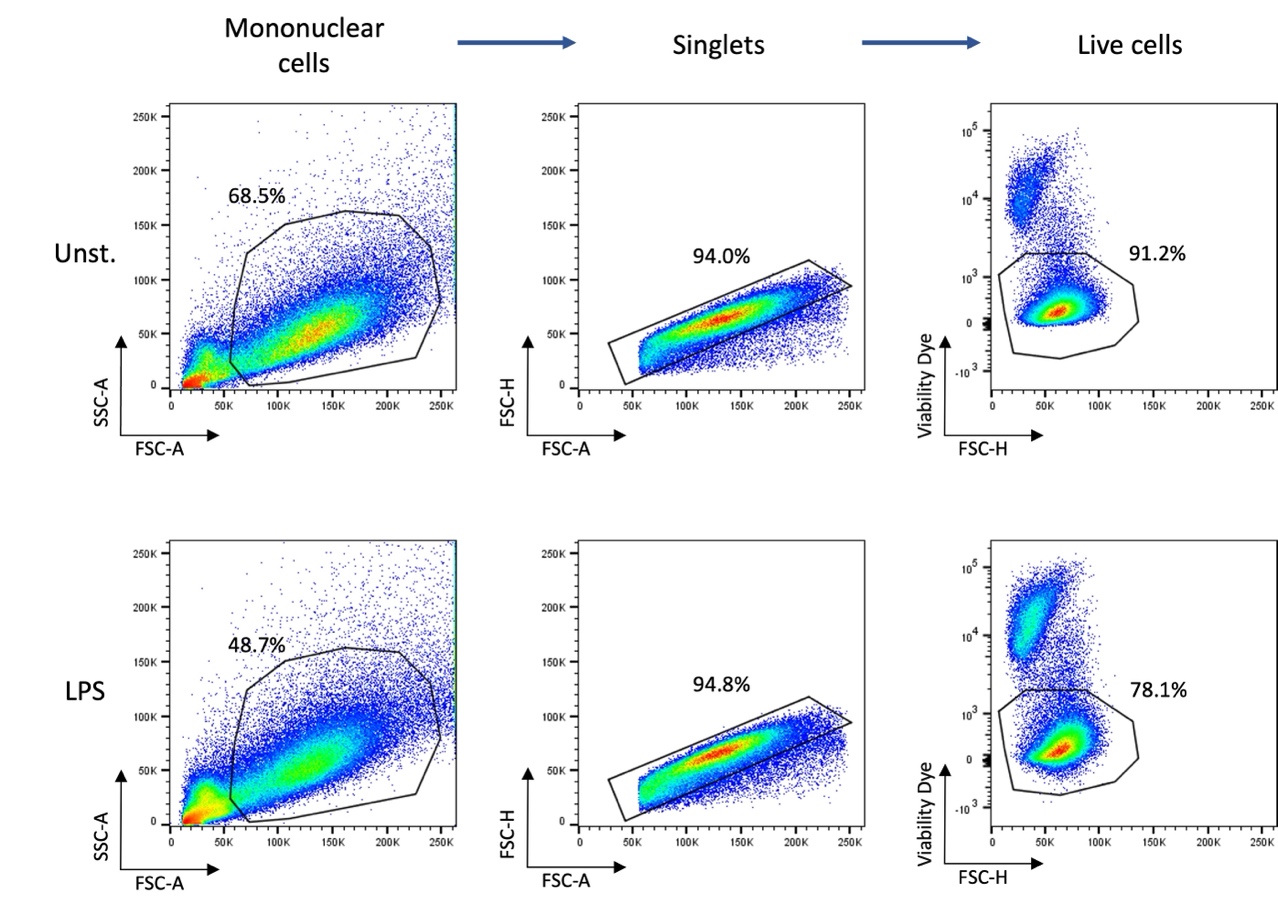


**Figure S3**. Gating strategy for flow cytometry analysis of MHCII, CD40, and CD80 expressions by unstimulated and LPS-stimulated chicken MoDCs.

The mononuclear cell population was gated based on FSC-A and SSC-A parameters, and singlets were selected from the FSC-A versus FSC-H. Dead cells were excluded using a viability dye.


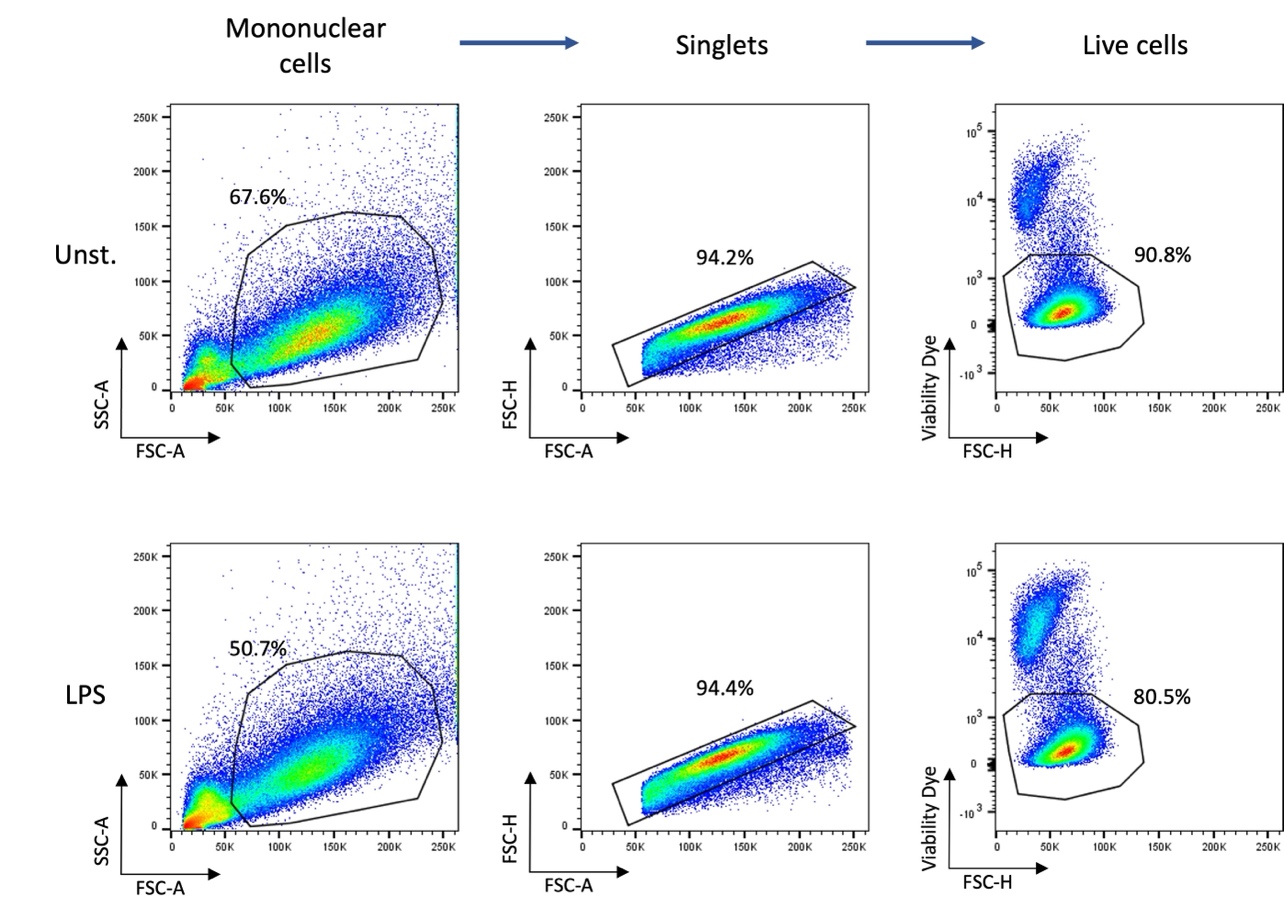


**Figure S4**. Gating strategy for flow cytometry analysis of MRC1LB and DEC205 expressions and FITC-Dextran capture by unstimulated and LPS-stimulated chicken MoDCs.

The mononuclear cell population was gated based on FSC-A and SSC-A parameters, and singlets were selected from the FSC-A versus FSC-H. Dead cells were excluded using a viability dye.


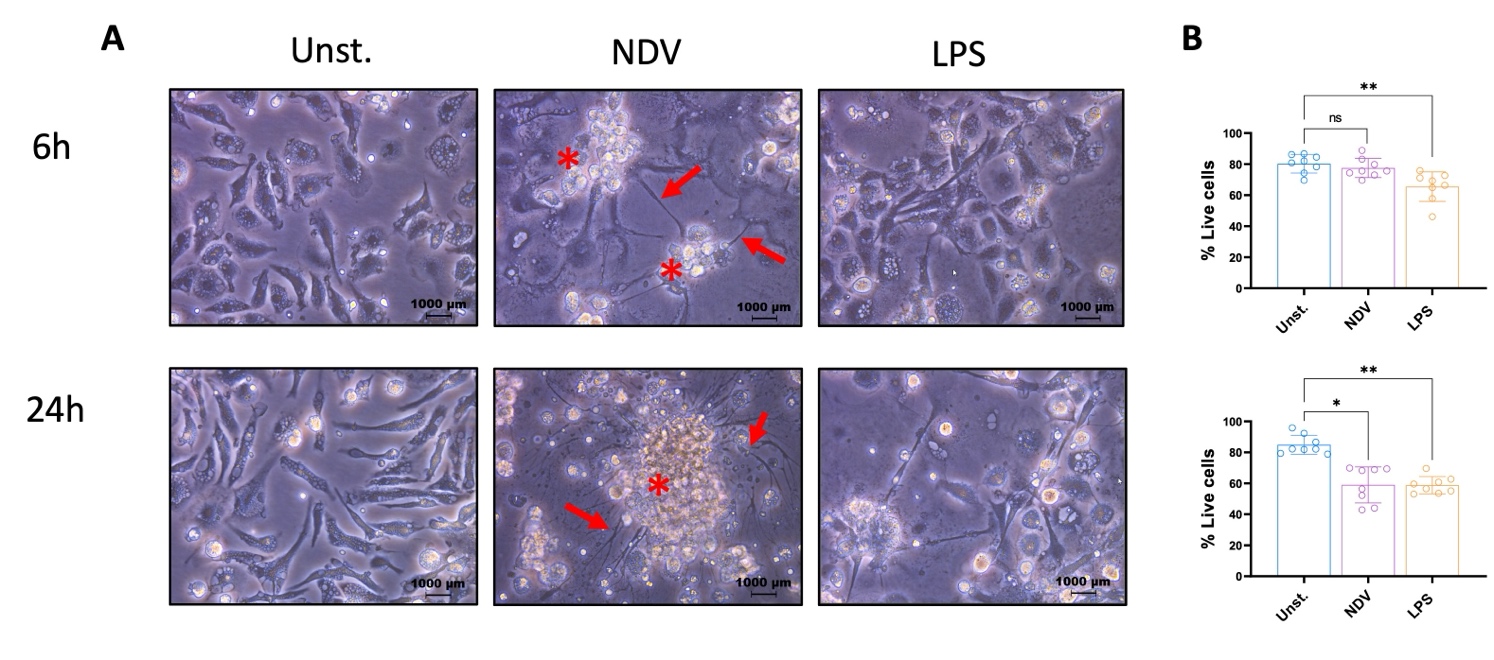


**Figure S5**. Morphology of chicken MoDCs stimulated with live NDV LaSota (NDV).

Chicken MoDCs were unstimulated or stimulated for 6 h or 24 h with 10 μg/mL NDV. Stimulation with 500 ng/mL LPS was used as the positive control. (A) Morphology under phase contrast microscopy of chicken MoDCs. The red arrows point to typical DC-like cytoplasm prolongations and red stars indicate loosely adherent aggregate cells. The scale bar indicates 1000μm. (B) The viability of MoDCs was examined by flow cytometry using the LIVE/DEAD® Fixable Near-IR Dead Cell Stain Kit. Plots representing the percentage of live cells among singlet cells after 6 h (upper panel) or 24 h stimulation (bottom panel) with live NDV are illustrated. Each circle refers to an individual chicken in the corresponding condition (n=8). One-way ANOVA and non-parametric Friedman test were used to determine statistical differences (*: p<0.05; **: p<0.01; ns, not significant).


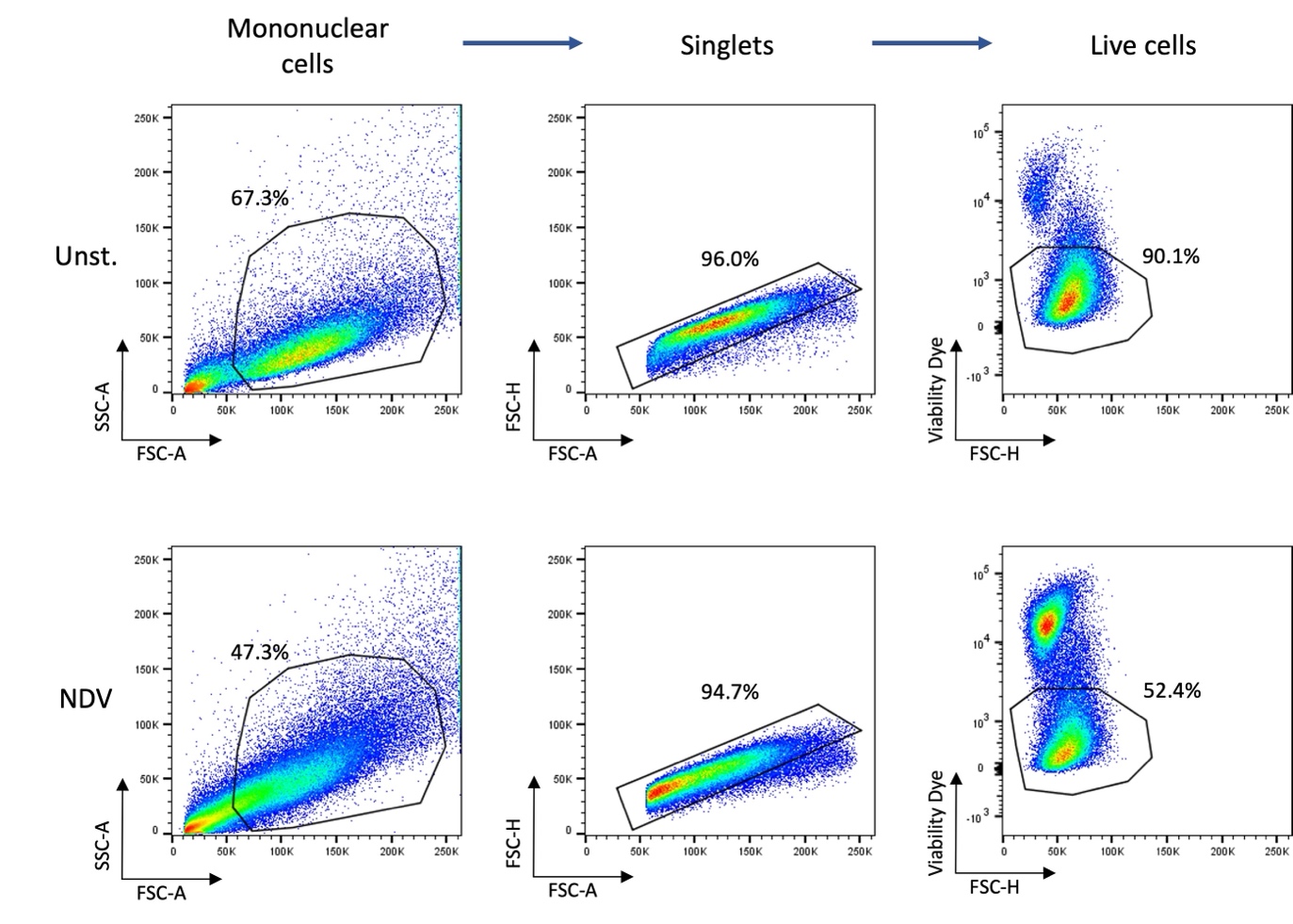


**Figure S6**. Gating strategy for flow cytometry analysis of MHCII, CD40, CD80, MRC1LB, and DEC205 expressions and FITC-Dextran capture by unstimulated and NDV-infected chicken MoDCs.

The mononuclear cell population was gated based on FSC-A and SSC-A parameters, and singlets were selected from the FSC-A versus FSC-H. Dead cells were excluded using a viability dye.


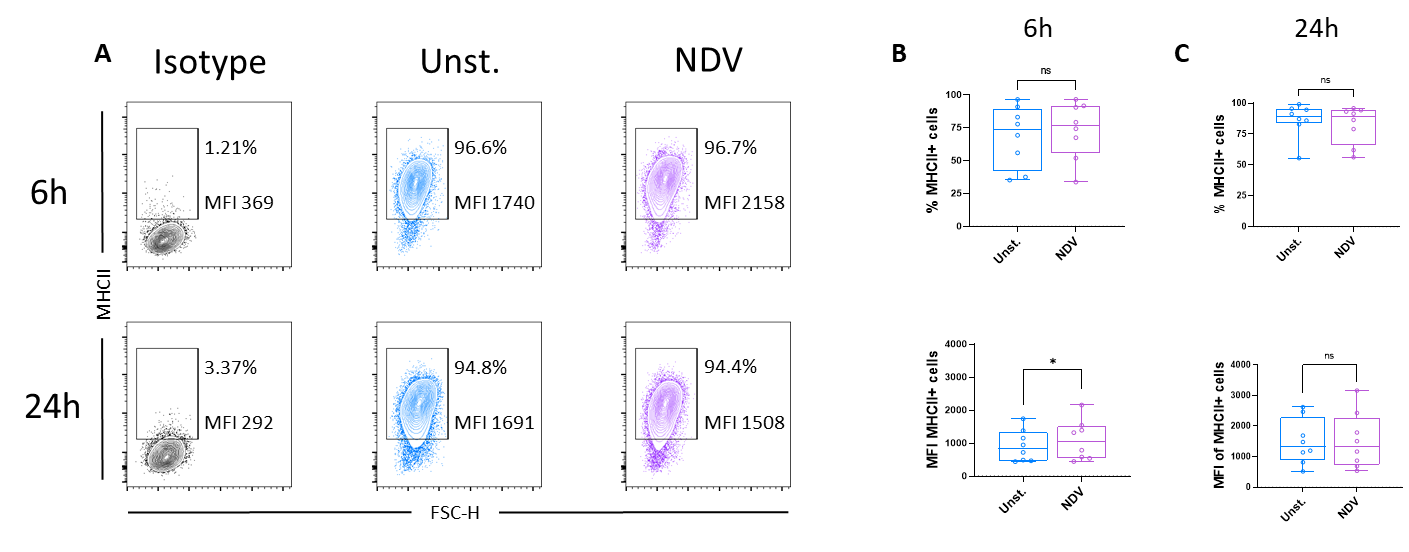


**Figure S7.**  6 h NDV stimulation slightly increases MHCII expression.

Analysis of MHCII expression in unstimulated and NDV-stimulated MoDCs after 6 and 24 h. (**A**) Representative contour plots of MHCII expression. The numbers represent the percentage of MHCII+ cells gated on viable cells and the MFI of MHCII+ cells. (**B**) Boxplot of the percentage of MHCII+ cells among viable cells after 6 h (upper panel) or 24 h (bottom panel) NDV stimulation. Each circle represents an individual chicken in the corresponding condition. A two-tailed paired t-test and a non-parametric Wilcoxon matched-pairs signed rank test were used to determine statistical differences (ns, not significant). (**C**) Boxplot of MFI determined on viable MHCII+ after 6 h (upper panel) or 24 h (bottom panel) NDV stimulation. Each circle represents an individual chicken in the corresponding condition. A two-tailed paired t-test was used to determine statistical differences (*: p<0.05; ns, not significant).


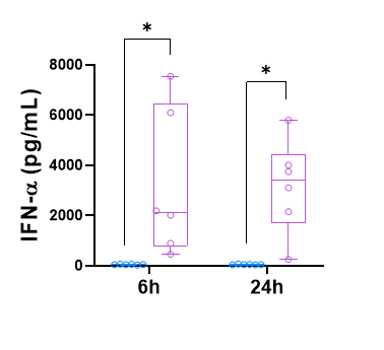


**Figure S8.** 6 h and 24 h NDV stimulation increases IFN-ɑ production.

The production of IFN-ɑ was quantified in the supernatants of unstimulated or NDV-stimulated MoDCs at 6 and 24 h by ELISA. Each circle represents an individual chicken. A two-tailed paired t-test and a non-parametric Wilcoxon matched-pairs signed rank test were used to determine statistical differences (*: p<0.05).
